# Impact of EcSOD Perturbations in Cancer Progression

**DOI:** 10.3390/antiox10081219

**Published:** 2021-07-29

**Authors:** Brianne R. O’Leary, Rory S. Carroll, Garett J. Steers, Jennifer Hrabe, Frederick E. Domann, Joseph J. Cullen

**Affiliations:** 1 Department of Surgery, University of Iowa College of Medicine, Iowa City, IA 52246, USA; brianne-oleary@uiowa.edu (B.R.O.); rory-carroll@uiowa.edu (R.S.C.); garett-steers@uiowa.edu (G.J.S.); jennifer-hrabe@uiowa.edu (J.H.); 2 Department of Radiation Oncology, University of Iowa College of Medicine, Iowa City, IA 52246, USA; frederick-domann@uiowa.edu; 3 Holden Comprehensive Cancer Center, Iowa City, IA 52246, USA; 4 Veterans Affairs Medical Center, Iowa City, IA 52246, USA

**Keywords:** reactive oxygen species (ROS), extracellular superoxide dismutase (EcSOD), cancer

## Abstract

Reactive oxygen species (ROS) are a normal byproduct of cellular metabolism and are required components in cell signaling and immune responses. However, an imbalance of ROS can lead to oxidative stress in various pathological states. Increases in oxidative stress are one of the hallmarks in cancer cells, which display an altered metabolism when compared to corresponding normal cells. Extracellular superoxide dismutase (EcSOD) is an antioxidant enzyme that catalyzes the dismutation of superoxide anion (O_2_^−^) in the extracellular environment. By doing so, this enzyme provides the cell with a defense against oxidative damage by contributing to redox balance. Interestingly, EcSOD expression has been found to be decreased in a variety of cancers, and this loss of expression may contribute to the development and progression of malignancies. In addition, recent compounds can increase EcSOD activity and expression, which has the potential for altering this redox signaling and cellular proliferation. This review will explore the role that EcSOD expression plays in cancer in order to better understand its potential as a tool for the detection, predicted outcomes and potential treatment of malignancies.

## 1. Reactive Oxygen Species (ROS)

Reactive oxygen species (ROS) are not only a normal byproduct of cellular metabolism that play a critical role in cell signaling processes, but also are generated by enzyme systems as an integral part of innate immune responses [1,2]. ROS are so named for their greater reactivities compared to molecular oxygen (O_2_) and receive attention for their toxic ability to damage proteins, lipids, and nucleic acids. Indeed, a delicate balance exists between ROS and their neutralizing antioxidant scavengers. An imbalance of ROS leads to pathological states. For example, an inability of phagocytes to generate ROS increases susceptibility to fungal and bacterial infections, as seen in chronic granulomatous disease [3]. Additionally, an overabundance of ROS, whether via increased production or a deficiency in antioxidants, leads to oxidative stress and has been linked to diverse pathologies, such as atherosclerosis, hypertension, ischemia/reperfusion injury, and neurodegenerative disease [4]. Increased oxidative stress has also been observed in cancer cells, which demonstrate altered metabolism as compared to normal tissues. This imbalance in the cellular redox state is associated with factors leading to malignant transformation, including altered cellular proliferation, stimulation of growth, and DNA damage [4,5].

Superoxide dismutases (SODs) are one of the most important defenses against oxidative damage. SODs consist of three antioxidant enzymes that catalyze the dismutation of superoxide anion (O_2_^−^) to hydrogen peroxide (H_2_O_2_), which is subsequently removed by catalase and peroxidases [6]. Each isoform catalyzes the same reaction yet is unique in its cellular location. Of the three SOD isoforms, CuZnSOD (SOD1) and MnSOD (SOD2) are located intracellularly; CuZnSOD is predominantly located in the cytosol and MnSOD in the mitochondria [6,7]. Extracellular superoxide dismutase (EcSOD), the third SOD discovered, is the predominant isozyme in extracellular fluids, such as plasma, lymph, ascites and cerebrospinal fluid [8]. It is a secreted enzyme that is located on the cell surface and extracellular matrix, with a small portion in extracellular fluids.

The net redox balance of the cell and its surroundings influences both normal and malignant cell behavior. As the only enzymes to dismutate superoxide, SODs play a significant role in this redox balance. However, the role that SOD plays in malignancy remains incompletely understood, particularly when looking at each isoform’s effect. Numerous studies have demonstrated that altering the redox balance via MnSOD overexpression (both in vitro and in vivo) decreases cell growth and survival and increases cell differentiation, consistent with the observation that MnSOD expression is frequently lost in tumor cells [5,9]. Similar results have been observed for CuZnSOD [5,9]. However, our understanding of how EcSOD affects tumor initiation, proliferation, and metastasis is in its early stages. This review is directed at summarizing the salient results of investigations, thus far on the role of EcSOD in cancer.

## 2. EcSOD

The EcSOD gene is localized in humans to chromosome 4q21 [10]. It shares 40–60% homology with SOD1, though virtually none with SOD2 [11]. EcSOD expression varies throughout the body. The highest concentrations are found in heart, lung, vascular smooth muscle, pancreas, and placenta, while low concentrations are in the liver, brain, and skeletal muscle [12,13,14,15,16]. The localization of EcSOD to the extracellular matrix (ECM) and cell surfaces is due to in part to an N-terminus targeting signal and its heparin binding domain (HBD) [17,18]. A targeting signal on the N-terminus of immature EcSOD instructs its secretion to the extracellular space [18]. Following secretion, the targeting signal is cleaved and the more stable mature EcSOD enzyme remains [18]. The HBD contributes a strong affinity for heparin and heparan sulfate, the latter of which is a critical component of cell surfaces and ECM. Nearly 99% of EcSOD is anchored to these heparan sulfate proteoglycans, with the remaining 1% located in the vasculature in equilibrium between the plasma and the endothelium [19]. EcSOD in vivo demonstrates heterogeneous affinity for heparin, due in part to post-translational proteolytic removal of the HBD [20,21]. The affinity for heparin varies across populations, in part because of single nucleotide polymorphisms (SNPs) within the HBD. One study of random blood donors identified that individuals with a HBD SNP demonstrate decreased heparin affinity with a subsequent ten-fold increase in plasma EcSOD levels [19]. In an animal model, such EcSOD variants with diminished or absent heparin affinities have significantly shorter half-lives (20 and 7 h, respectively, versus 85 h for unaltered EcSOD) [22].

### 2.1. EcSOD Expression

Factors affecting the regulation of EcSOD expression are numerous and varied. Some of the major factors include nitric oxide, which can be inactivated by superoxide anion and has been shown to upregulate EcSOD in a mouse model [23]. Angiotensin II has also been shown to amplify EcSOD expression in mice by increasing transcription as well as providing greater stabilization (and thus longer half-life) of EcSOD mRNA [24]. Additionally, stimulating EcSOD expression is heparin and, to a lesser extent, heparan sulfate as demonstrated in human fibroblasts [25]. Cytokines were found to influence EcSOD expression in human dermal fibroblasts and vascular smooth muscle cells. In both cell types, IFN-γ increased, whereas TNFα decreased the expression of EcSOD [26]. TGFβ decreased EcSOD in fibroblasts, whereas IL-4 increased EcSOD in smooth muscle cells [12,26]. Hormones also influence EcSOD expression. In rat vascular smooth muscle cells, as well as in human monocytes, treatment with estrogen significantly increased EcSOD expression [27]. Copper, which is a required cofactor for EcSOD activity, has also been demonstrated to positively regulate EcSOD transcription, leading to increased EcSOD mRNA and protein. This effect is mediated through the copper chaperone Antioxidant-1 [28].

### 2.2. EcSOD and Disease

Aberrations in EcSOD expression have been associated with a variety of human diseases. EcSOD plays a critical role in a variety of human diseases and, generally, loss of EcSOD confers greater disease activity while restoration or overexpression can ameliorate those processes. In vascular diseases, the expression of EcSOD often confers a protective effect. Plasma EcSOD expression and coronary artery disease, as documented by angiography, demonstrated that low plasma EcSOD was an independent risk for a history of myocardial infarction (MI) and that higher EcSOD levels were associated with MIs occurring at a later stage [29]. Using EcSOD-deficient mice, two studies demonstrated that the administration of angiotensin II (Ang II) caused a greater degree of hypertension in the EcSOD-deficient mice than in the controls, and that the presence of EcSOD decreased blood pressure, decreased superoxide, and enhanced the relaxation of vessels [30,31]. Additionally, EcSOD overexpression was shown to protect against the development of pulmonary hypertension in mice, while treatment with EcSOD improved established pulmonary hypertension [32]. In a lamb model of persistent pulmonary hypertension of the newborn, decreased EcSOD activity was noted in pulmonary artery smooth muscle cells relative to the controls. The EcSOD activity decrease was thought to be mediated by H_2_O_2,_ generated by persistent pulmonary hypertension and hyperoxia [33].

The lung is one of the organs with the highest EcSOD expression [12,13,14]. Given the lung’s constant exposure to inhaled ROS, EcSOD is poised to play a critical role in protecting lung tissue. EcSOD’s ability to protect the ECM against oxidative stress was examined using EcSOD-deficient and EcSOD-overexpressing mice. Mice were administered cigarette smoke, which is known to induce the endogenous production of ROS. EcSOD-deficient mice developed enlarged airspaces and impaired lung function and exercise capacities, which were accompanied by increased extracellular matrix fragmentation. Conversely, EcSOD-overexpressing mice had less severe emphysematous lung changes and were protected against oxidative fragmentation of the extracellular matrix [34].

### 2.3. EcSOD and Cancer

**Figure 1:**
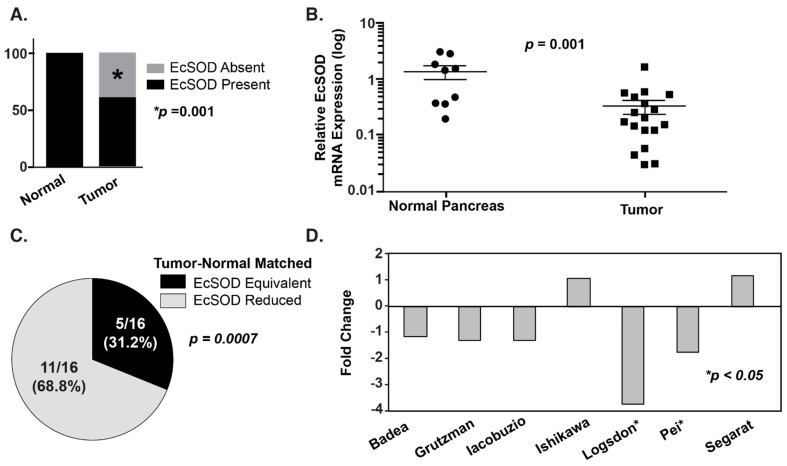
EcSOD expression is significantly decreased in pancreatic ductal adenocarcinoma (PDA) relative to normal pancreatic ductal epithelium. (**A**) EcSOD expression was evaluated by immunohistochemistry (IHC). EcSOD expression was scored as present or absent. All core biopsies from normal pancreatic ductal epithelium, 28/28 (100%), had high EcSOD expression, while 18/44 core biopsies from PDA lacked EcSOD expression (41%). *p* = 0.001. (**B**) EcSOD mRNA expression is significantly reduced in tumor compared to normal pancreas. The data were normalized to β-actin expression and are presented as fold-change (log scale) of tumor samples compared to normal pancreas. (**C**) IHC was performed on surgical specimens from PDA patients. Areas of normal pancreas showed intact EcSOD expression in all cases, while malignant ductal epithelium showed reduced EcSOD expression in 11/16 specimens *p* = 0.0007. (**D**) Analysis of existing Oncomine data revealed a consistent decrease in EcSOD mRNA expression relative to normal pancreas in 5/7 studies; 2 studies were highly significant [39].

Given its extracellular location and its distribution throughout the body, EcSOD may play a role in cancer-related processes, such as cell signaling, angiogenesis, and perturbations in the extracellular matrix [5]. EcSOD expression affects the development and progression of malignancies with loss of EcSOD expression having been demonstrated both in vitro and in vivo [35,36,37,38,39] (Figure 1A–C). The greatest evidence for this is in lung and pancreatic cancer, where low expression of EcSOD in adenocarcinoma cell lines and human cancer specimens has been demonstrated compared to normal tissue [35,36,37,39]. Analyses of human non-small cell lung cancer tissue specimens have borne out this association. EcSOD immunoreactive protein expression has been found to be decreased by 70–90% in squamous cell and adenocarcinoma tumor samples as compared to normal adjacent tissue [35,36,37]. Similarly, the vast majority of surveyed tumor samples show no immunohistochemical staining for EcSOD [35]. Furthermore, EcSOD appears to be lost early in lung cancer progression. A tissue array of 40 lung tumors of varying histology and grades demonstrated that EcSOD mRNA expression was significantly decreased compared to normal lung tissue, and the level of EcSOD mRNA progressively decreased with advancing grade [37]. In both lung adenocarcinoma cell lines and tissue samples, EcSOD appears to be silenced at the transcriptional level, with low mRNA expression corresponding to decreased protein expression [37]. This phenomenon has since been shown in both pancreatic (Figure 1) and mammary adenocarcinoma samples with similar results [38,39].

In an attempt to identify an association between expression of oxidative stress modifying genes (including EcSOD) and breast cancer prognosis, transcript levels of gene expression in 120 tumor and 51 paired, adjacent non-neoplastic tissues demonstrated that EcSOD expression was significantly downregulated in tumor tissue compared to adjacent normal specimens. Similar to the findings in lung cancer, EcSOD expression was higher in Grade I/II tumors versus Grade III tumors, suggesting a progressive loss of EcSOD expression with higher tumor grade. No association was found between patient’s age at diagnosis, tumor size, or histological type [40]. These findings are similar in both thyroid cancer [41,42] and renal cell cancer [43,44]. A DNA array of human samples containing normal thyroid, papillary thyroid cancer, and anaplastic thyroid cancer showed significantly lower EcSOD expression in the cancer samples compared to normal [41]. Loss of EcSOD expression induced oncogene-mediated transformation and the de-differentiation of rat thyroid cells [41]. In human renal cell carcinoma samples, EcSOD showed the lowest amount of immunoreactivity compared to other antioxidant enzymes studied, which likely represents loss of expression compared to the abundant expression in normal renal tissue [43,44].

### 2.4. Hypermethylation of EcSOD

**Figure 2:**
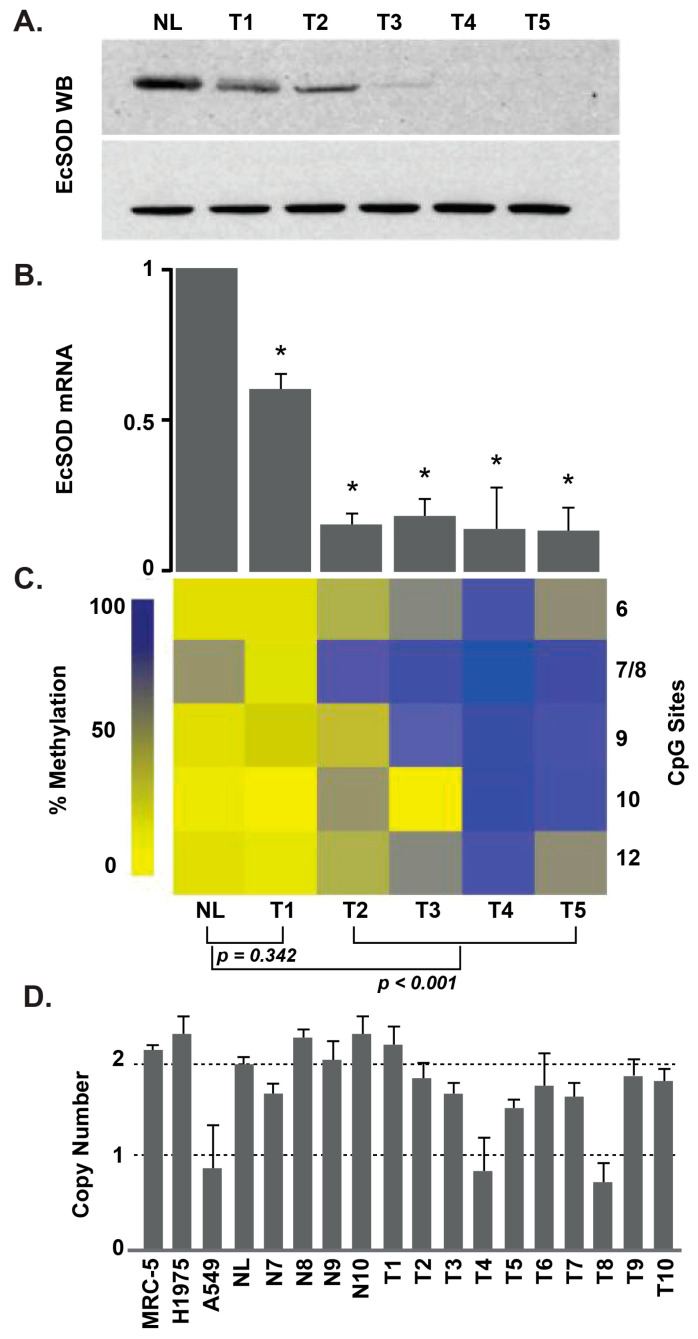
EcSOD protein and mRNA expression are decreased in lung adenocarcinoma, with frequent alterations in EcSOD promoter methylation. (**A**) Western blot for EcSOD. NL represents normal lung tissue while LT1-LT5 are lung adenocarcinoma tumor samples. The x axis represents the clinical sample labels for both panels A and B. (**B**) Expression of EcSOD mRNA, as determined by quantitative real-time RT-PCR. Each value is shown as a relative value compared to NL and normalized to 18S transcription level (* *p* < 0.05 vs. normal). (**C**) Heat map depicting the percent methylation of CpG sites 6–12 (right y axis) of the EcSOD promoter in NL and LT1-LT5. As depicted in the legend along the left y axis, low methylation is labeled in light yellow and the highest methylation in dark blue. (**D**) Results of TaqMan copy number variation assay in human lung cancer samples and cell lines. The y axis represents gene copy number, and the x axis represents clinical samples. Error bars, S.D. of each sample run in triplicate [37].

An understanding of why EcSOD expression is so low in many cancers has been sought. In a study of both lung adenocarcinoma cell lines, as well as human lung cancer samples, an epigenetic mechanism behind low EcSOD expression was examined (Figure 2A,B). The investigators found that the methylation status of the EcSOD promoter inversely corresponded to EcSOD expression [37] (Figure 2C). That is, significantly higher cytosine methylation in the EcSOD promoter regulatory region corresponded to cells and tumor samples with decreased EcSOD [37] (Figure 2D). Similar epigenetic modifications were found in breast cancer, where a significant increase in the methylation of the EcSOD promoter in patients with breast cancer compared to controls was identified [45,46]. In another study, combined bisulfite restriction analyses of in vivo tumor samples demonstrated an association of DNA methylation with the loss of EcSOD expression [38].

### 2.5. Single Nucleotide Polymorphisms

Single nucleotide polymorphisms (SNPs) have been described for EcSOD. At least one variant has been associated with altered outcomes in human diseases. Similar attempts to link SNPs to cancer risk have been undertaken, with varying results. The possibility of a link between genetic polymorphisms in EcSOD and pancreatic cancer risk was examined. This case–control study compared EcSOD polymorphisms at rs1799895 (Arg231Gly) of 235 pancreatic cancer patients to 265 controls, but failed to demonstrate any concordance between polymorphism and cancer risk [47]. Similarly, two separate studies of SNPs and prostate cancer did not yield any link for polymorphisms at rs8192287, rs699473, rs17878863, rs17881426, rs1007991, rs8192291, rs2695232, rs1799895, rs2853796, and rs2855262 [48,49]. However, in breast cancer, a comparison of EcSOD genotype and clinical data of patients with breast cancer revealed several important associations. First, patients with the threonine allele in SNP rs2536512 had a significantly greater rate of estrogen receptor positive tumors than patients carrying the Ala/Ala genotype. Secondly, the SNP rs699473 was associated with modified progression-free survival; those patients carrying the T allele had significantly poorer progression-free survival than patients carrying the CC (homozygous normal) genotype. This was true also in the group of patients treated with hormonal therapy [40]. This same SNP was implicated in an increased risk of adult brain tumors. In a case–control study, the investigators detected an increased risk of both glioma and meningioma in patients with the C variant (CT genotype) of SNP rs699473 [50].

### 2.6. Phenotypic Effects of EcSOD Expression

**Figure 3:**
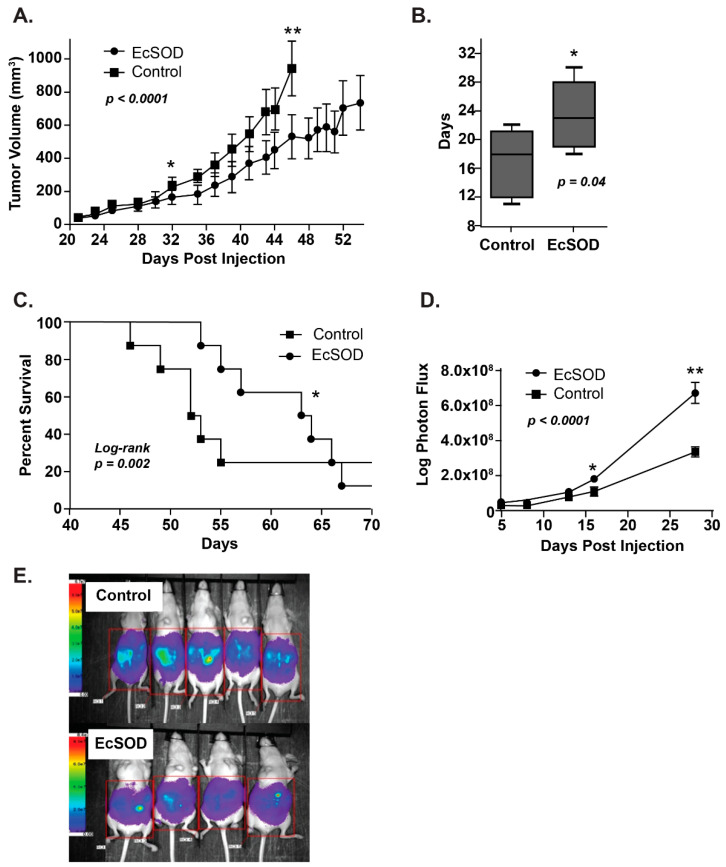
Overexpression of EcSOD reduces tumor xenograft growth and peritoneal growth and increases doubling time and animal survival in athymic nude mice. (**A**) EcSOD overexpressing-tumor volumes were significantly smaller (** *p* < 0.05 vs. EcSOD) starting on day 32. (**B**) EcSOD tumors demonstrated a significantly longer doubling time compared to the control (* *p* < 0.05 EcSOD vs. control). (**C**) Kaplan–Meier survival plot demonstrates that mice baring EcSOD, expressing BxPC3 tumors had prolonged median survival compared to mice baring empty vector tumors (63 vs. 52 days * *p* = 0.002, log-rank). (**D**) Intraperitoneal luciferase-expressing EcSOD tumors demonstrated decreased growth measured by total photon flux when compared to empty vector control tumors. (**E**) Representative image of bioluminescence imaging of mice 28 days post-intraperitoneal injections of luciferase-expressing Bx-EcSOD or Bx-Control cells. Mice injected with luciferized Bx-EcSOD cells showed statistically less tumor burden than control mice [39].

As noted above, EcSOD expression is lost in a number of tumor types, thus reintroducing EcSOD may affect tumor growth and progression. To that end, several investigators have examined the effects of EcSOD overexpression both in vitro and in vivo (Table 1). Pancreas cancer cells forced to overexpress EcSOD demonstrate a dose-dependent decrease in cell growth and clonogenic survival compared to parental cells [39,51,52]. The in vivo effects of EcSOD overexpression with both adenoviral and stable cell constructs were tested in mouse models. Animals treated with EcSOD had the smaller tumor volumes, due to significantly slowed growth rate and increased survival rate compared to controls [39,51,52] (Figure 3A–C). The overexpression of EcSOD was also found to decrease peritoneal growth in an intraperitoneal metastasis model [39]. Cells stably expressing EcSOD injected via intraperitoneal injection formed significantly less peritoneal tumor growth over time [39] (Figure 3D,E).

Similar findings were demonstrated in both melanoma and breast cancer [38,59]. Mice administered adenovirus to overexpress EcSOD had an 80% inhibition of melanoma tumor growth compared to controls. Further findings were obtained when cells were first transduced to overexpress EcSOD and then injected into untreated mice, demonstrating the effect of EcSOD on tumor growth [38,59]. In breast cancer, both experimental lung and spontaneous metastasis models demonstrated that overexpression of EcSOD through adenoviral vectors resulted in reduced lung metastasis compared to controls. Mice injected with adenoviral EcSOD in both models also displayed increased median survival rates [38]. Similar results were produced using an SOD3-mimetic, polynitroxyl-albumin, which increased survival and decreased lung metastasis in mice with triple-negative breast cancer flank tumors [62]. Consistent with these findings, forced overexpression of EcSOD in lung and pancreas cancer cell lines reduced the malignant phenotype by increasing cell doubling times and decreasing clonogenic survival [37,39,57]. These changes were even more pronounced in breast cancer cells treated with an adenoviral EcSOD construct containing a deletion in the HBD, which eliminated the ability of EcSOD to associate with heparan sulfate and, therefore, its sequestration on the cell surface. The mechanism by which EcSODΔHBD would impair cancer growth more than the full-length construct are not yet elucidated but may relate to greater bioavailability of the unbound EcSOD.

While much of the work with EcSOD is related to the effects on cell proliferation, EcSOD also has been implicated in helping cells survive in a quiescent state. As quiescent cells have been found to be relatively resistant to apoptosis, an understanding of the mechanisms by which they maintain survival in the quiescent state may be important. One mechanism the cells may be using to maintain cell viability while in a resting state is via reduction in oxidative stress. Mirk/Dyrk1B, a serine/threonine kinase activated by oncogenic K-*ras*, was found to be most active in quiescent pancreatic cancer cells [53]. Mirk was shown to affect quiescent cell viability in part by reducing oxidative stress through the increased transcription of several antioxidant genes including EcSOD [53].

Ovarian cancer cells become sensitive to apoptosis through manipulations of the redox balance [60]. The role of EcSOD on ovarian cancer cell survival is only beginning to be understood. Two studies have examined its role in apoptosis and have demonstrated varying relationships between EcSOD expression and apoptosis. First, the inhibition of dehydrogenase kinase induced a shift from glycolysis to oxidative phosphorylation, resulting in an increase in EcSOD mRNA and protein as well as an increased rate of apoptosis [63]. Secondly, the inhibition of ROS-producing enzymes, such as NADPH oxidase also resulted in decreased EcSOD and increased apoptosis [61]. These findings demonstrate that the complex relationship between EcSOD and apoptosis in ovarian cancer requires further investigation.

The role of EcSOD in the evasion of apoptosis was indirectly examined in prostate cancer cells. Lens epithelium-derived growth factor (LEDGF) is overexpressed in prostate cancer and promotes resistance to cell death. A PCR array examining antioxidant gene expression changes in response to alterations in LEDGF expression revealed corresponding changes to levels of EcSOD. Thus, EcSOD may be one of several genes regulated by LEDGF and may play a role in prostate cancer cell survival and resistance to oxidative stress-induced cell death [55].

A critical part of tumor growth and proliferation is angiogenesis. In in vivo tumor models, as well as in vitro assays, EcSOD significantly affected vascular endothelial growth factor (VEGF) [54,59]. Mice treated with an adenoviral vector to overexpress EcSOD in both melanoma and pancreatic tumors showed significantly decreased VEGF expression on immunohistochemistry and Western blot as well as decreased tumoral vessel density on hematoxylin and eosin and immunostaining [54,59]. Both breast and pancreatic cancer cells with forced overexpression of EcSOD also demonstrated a significant decrease in VEGF [54,57]. Contrary to the above results, a recent study in breast cancer progression showed that the restoration of EcSOD in VEGF-C knockdown cells actually increased tumor progression and metastases in vivo. The investigators concluded that EcSOD was a mediator of VEGF-C-induced metastasis in this specific murine cell line [64]_._ EcSOD also effects tumor vasculature through interaction with HIF-2α [65,66]. Mira et al. argued that increased EcSOD expression increased chemotherapy delivery to tumors by stabilizing HIF-2α, leading to increased vascular endothelial cadherin which reduces vessel leakage [65]. The EcSOD interaction with HIF-2α also improved T cell tumor infiltration in an in vivo thymoma model [66].

**Figure 4:**
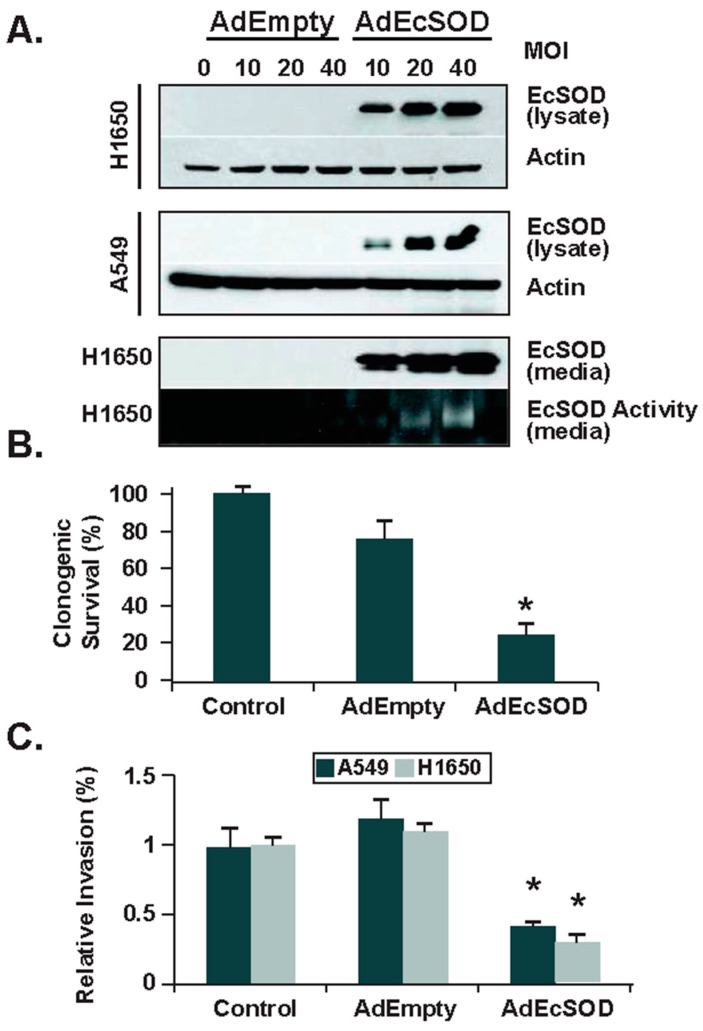
Forced re-expression of EcSOD reduced the clonogenic capacity and invasive potential of human lung cancer cells. (**A**) Following adenovirus transduction of EcSOD (AdEcSOD) in lung cancer cell lines, Western blot shows increased expression of EcSOD in both cell lysates and in culture media. An activity gel (bottom panel) shows that EcSOD was secreted and was catalytically active in the adenovirus infected H1650 cells. Empty vector infection did not induce EcSOD expression. (**B**) Clonogenic survival is significantly decreased (* *p* < 0.05 vs. AdEmpty) after forced EcSOD re-expression. (**C**) Invasion through Matrigel was assessed after forced EcSOD re-expression [37].

The ability of tumor cells to invade surrounding tissues is critical to their ability to metastasize. In addition to affecting tumor growth and angiogenesis, EcSOD has been demonstrated to modify cancer cell invasive capacity. Separate in vitro models of forced EcSOD overexpression in prostate, breast, lung, and pancreatic cancer cells all demonstrated a significant reduction in the cells’ ability to invade [37,39,56,57] (Figure 4). Mechanisms behind this reduced invasive ability may be related to alterations in matrix metalloprotein (MMP), heparanase expression, or redox imbalances in the absence of EcSOD. In the prostate cancer model, decreased invasiveness in EcSOD overexpressing cells was accompanied by decreased MMP protein expression activity [56]. Heparanase, which degrades extracellular matrix components is upregulated in many cancers. The breast and lung models suggested that the effect of EcSOD on invasive capacity may be related to heparanase expression, where cancer cells overexpressing EcSOD had diminished heparanase promoter activity, decreased heparanase transcription and activity, and increased intact heparan sulfate on cell surfaces [37,57]. This effect may be mediated through NF-κB, which has been shown to correlate with heparanase expression in several cancers, as the overexpression of EcSOD both in vitro and in vivo was associated with decreased NF-κB expression and nuclear localization [37,59]. Pancreatic cancer models point to redox imbalances as a possible mechanism of invasive potential. Incubation with N-acetylcysteine (non-specific thiol antioxidant) or N^G^-nitro-L-arginine (competitive inhibitor of NO-synthase activity) significantly reduced invasion in control cells without impacting EcSOD overexpressing pancreatic cells [39]. Alternatively, the incubation of pancreatic ductal adenocarcinoma cells with catalase, an antioxidant enzyme that scavenges H_2_O_2_, did not decrease the invasion of either control or EcSOD overexpressing cells [39]. These combined results suggest that reduced invasion is not due to the accumulation of extracellular H_2_O_2_ but may instead be due to EcSOD’s ability to remove superoxide (O_2_^−^), thereby limiting the formation of peroxynitrite (ONOO^−^) in the extracellular space. Similar in vivo findings are presented with the use of lecithinized superoxide dismutase (PC-SOD) in pulmonary metastasis of mice, where it was concluded that PC-SOD caused a dose-dependent decrease in metastatic activity through a mechanism that might involve the inhibition of ONOO^−^ formation [67].

Evidence for the role of nitric oxide (NO), (O_2_^−^), and ONOO^−^ in metastatic potential has previously been widely discussed. Increased expression of nitrotyrosine, a biomarker for peroxynitrite, has been shown in both pancreatic adenocarcinoma and esophageal squamous cell carcinoma when compared to normal tissue [68,69]. Detection of nitrotyrosine in esophageal cancer was associated with increased tumor invasion, occurrence of metastasis, pathological stage and decreased survival [69]. Similar findings were recently determined in resected pancreatic adenocarcinoma patient samples where a loss of EcSOD expression was observed alongside an increase in immunoreactive nitrotyrosine [39]. These results highlight the complicated nature of metastasis and suggest that EcSOD may help elucidate the factors that lead to disease progression.

## 3. SOD Mimetics in Cancer Therapy

As decreased levels of EcSOD have been implicated in many cancers, strategies to boost superoxide scavenging with SOD mimetics have been pursued as cancer therapy. SOD mimetics, synthetic compounds that mimic the activity of native SOD, have been studied as adjuvants for inhibiting cancer cell growth and as radioprotectants for normal tissues. For example, Chatterjee et al. showed that an SOD mimetic in the class of Manganoporphyrins (MnPs), MnTE-2-PyP, enhanced tumor radiosensitivity in an in vivo prostate cancer model. This same molecule was also found to protect normal pelvic tissue from radiation damage [70,71]. Another SOD mimetic, MnTnHex-2-PyP^5+^, also demonstrated promising radioprotective effects by reducing lung fibrosis after whole-thorax irradiation in a non-human primate model [72]. SOD mimetics have also been found to be safe in human clinical trials. In a Phase IIb trial, the SOD mimetic GC4419 significantly reduced severe oral mucositis in patients undergoing radiation therapy for head and neck cancer [73].

### SOD Mimetics and Pharmacological Ascorbate

Pharmacological ascorbate (P-AscH^−^, high dose intravenous Vitamin C) is a promising adjuvant for pancreatic cancer therapy [74,75]. P-AscH^−^ achieves plasma concentrations of ascorbate in the 10–20 mmol range, compared to 0.2 mmol plasma levels when ascorbate is administered orally. It is at these elevated levels that P-AscH^−^ is selectively cytotoxic to cancer cells via generation of H_2_O_2_ and has proven to be a safe adjuvant to standard of care therapy in our clinical trials [75,76] Interestingly, SOD mimetics have been shown to enhance the cytotoxic effects of P-AscH^−^ in pancreatic cancer [77]. Although MnPs were originally developed as SOD mimetics, they act as superoxide reductases in the presence of P-AscH^−^ as the central Mn (III) is reduced to Mn (II) by P-AscH^−^. Mn (II) then reacts with O_2_, forming superoxide, which then becomes H_2_O_2_ and O_2_ [78]. MnPs have varying degrees of physicochemical properties when combined with P-AscH^−^. In one study, MnT4MPyP had the greatest effect on increasing P-AscH^−^ oxidation rates when compared to two other MnPs [79]. This interaction between P-AscH^−^ and MnPs synergized to decrease clonogenic survival in PDAC cells. In another study, MnT4MPyP not only increased P-AscH^−^ oxidation in vivo, acting as a radiosensitizer to pancreatic cancer cells, it also increased P-AscH^−^ oxidation ex vivo when added to plasma samples of patients who had undergone P-AscH^−^ infusion during radiotherapy [77]. The aforementioned mimetic, GC4419, is a pentaazamacrocyclic Mn (II)-containing compound. GC4419 also increases P-AscH^−^ cytotoxicity to cancer cells by increasing the oxidation rate of P-AscH^−^ and H_2_O_2_ generation. When GC4419 is combined with P-AscH^−^, human lung and head and neck cancer cells are sensitized to radiation [80].

**Figure 5:**
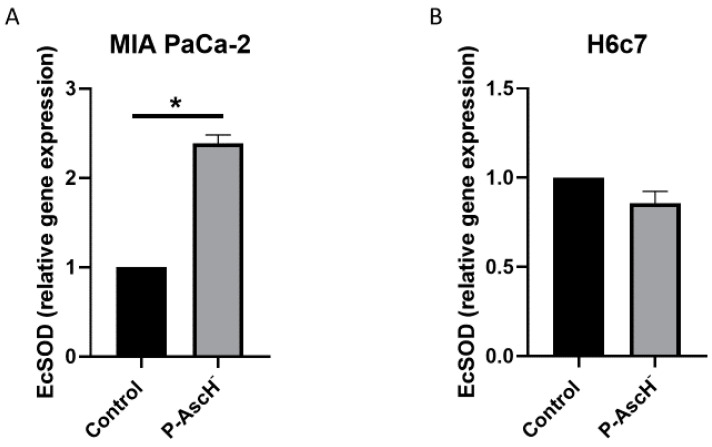
P-AscH^-^ increases EcSOD expression in PDAC. (**A**) MIA PaCa-2 cells were treated with P-AscH^−^ (10 pmole/cell, 1 mM for 1 h). Expression of EcSOD mRNA determined by quantitative real-time RT-PCR 48 h after P-AscH^−^ treatment. MIA PaCa-2 cells treated with P-AscH^−^ had increased expression relative to control and normalized to 18s expression, * *p* < 0.01, *n* = 3. Two-tailed Student’s t-test was performed. (**B**) H6c7 cells showed no difference in EcSOD expression 48 h after P-AscH^−^ treatment (10 pmole/cell, 1 mM for 1 h), *p* = 0.09, *n* = 3. Two-tailed Student’s t-test was performed.

Because of these encouraging interactions between P-AscH^−^ and SOD mimetics, our group has investigated the relationship between P-AscH^−^ and native EcSOD expression. We recently discovered that P-AscH^−^ treatment increases the expression of DUOX1 and DUOX2, members of the NADPH oxidase family, in pancreatic cancer cells [81]. These enzymes, similar to EcSOD, have decreased expression in pancreatic cancer cells compared to normal pancreatic cells. In pancreatic cancer cells, P-AscH^−^ treatment (10 pmole/cell, 1 mM for 1 h) increased EcSOD expression 48 h later after treatment, with no increases in normal pancreatic epithelial cells (Figure 5). These results demonstrate that P-AscH^−^, in addition to its direct cancer-specific cytotoxicity, may induce increased expression of EcSOD and other potential tumor suppressor genes by direct stimulation or through epigenetic mechanisms. The elucidation of these pathways may yield promising data for both understanding the ROS and EcSOD relationship with cancer as well as provide novel therapeutic options for future cancer patients.

## 4. Summary

The role of EcSOD in cancer development and progression demonstrates several themes. First, EcSOD expression is typically lost in malignant transformation, and the cause of loss often appears to be epigenetic, due to the aberrant methylation of the EcSOD promoter. Secondly, reinstating EcSOD expression reduces the malignant phenotype, including slowing cell growth and division, impairing their secretion of pro-angiogenic factors, and inhibiting invasion. Reducing these malignant phenotypes by increasing EcSOD with mimetics, porphyrins, or P-AscH^−^ is a promising strategy for cancer therapy development. Our ability to understand how perturbations in EcSOD expression relates to the development of cancer may facilitate our ability to detect and treat malignancies.

## Figures and Tables

**Table 1:** EcSOD in malignancy.

Tissue	Growth/Proliferation	Cell Types	Model	Other Effects	Reference
Pancreas	Decreased doubling time, tumor growth	Mia PaCa-2, BxPC-3	In vitro—adenoviral overexpression, In vivo—intratumoral injection with adenoviral constructs		Teoh/Cullen 2007 [52]
Pancreas	Decreased doubling time, tumor growth	Mia PaCa-2, H6c7	In vitro—adenoviral overexpression, In vivo—intratumoral injection with adenoviral constructs		Du/Cullen 2012 [51]
Pancreas	Increased indirectly; survival of quiescent cells	SU86.86, Panc-1	In vitro	Loss of EcSOD correlated with decreased survival of quiescent cells	Deng 2009 [53]
Pancreas	Decreased doubling time, tumor growth	Mia PaCa-2, BxPC-3	In vitro*—*stable cell overexpression, In vivo—subcutaneous injection with stably overexpressing cells	EcSOD overexpression decreased invasive capacity. Loss of EcSOD correlated with worsened disease biology	O’Leary 2015 [39]
Pancreas	Decreased tumor growth	Mia PaCa-2	In vivo*—*intratumoral injection with adenoviral constructs	EcSOD overexpression suppressed VEGF levels	Sibenallar 2014 [54]
Prostate	NA	PC3	qPCR gene microarray	EcSOD expression increased/decreased corresponding to LEDGF expression	Basu 2011 [55]
Prostate	NA	DU145, PC-3, WPEI-NB26	In vitro—adenoviral overexpression	Overexpressed EcSOD decreased cell invasiveness	Chaiswing 2008 [56]
Breast	Decreased doubling time, clonogenic survival	MDA-MB231, MDA-MB 435	In vitro*—*adenoviral overexpression	Decreased invasive capacity (matrigel)	Teoh/Domann 2009 [57]
Breast	NA	Human breast tissue samples		Increase in hypermethylation of EcSOD promoter in breast ca	Naushad 2011 [45]
Breast	NA	Human breast tissue samples		Decreased EcSOD in tumor vs. normal; higher expression in lower grade tumor samples	Hubackova 2012 [40]
Breast	NA		Decreased EcSOD in mRNA and protein in tumor vs. normal; inverse correlation with clinical stages of cancer		Teoh 2014 [38]
Breast	NA	MDA-MB231, MDA-MB468, RMF	In vitro—MDA-MB231 overexpressing EcSOD cell line	EcSOD overexpression suppresses oncogenic cancer-fibroblast interaction	Golden 2017 [58]
Breast	NA	Human breast tissue samples	Pyrosequencing analysis in breast carcinoma samples	Increase in methylation status of EcSOD promoter in tumor vs. normal tissue	Griess 2020 [46]
Lung	Decreased clonogenic survival	HAE, A549, MRC-5, NCI-H1975 & H1650; lung tissue samples	In vitro—adenoviral overexpression	Decreased invasive capacity (matrigel)	Teoh 2012 [37]
Lung	NA	Human lung ca tissue samples		Decreased EcSOD in tumor vs. normal	Yoo 2008 [36]
Lung	NA	Human lung ca tissue samples		Decreased EcSOD in tumor vs. normal	Svensk 2004 [35]
Melanoma	No change	B16-F1	In vitro—adenoviral overexpression; In vivo—tumor xenografts	Decreased tumor size with EcSOD overexpression.	Wheeler 2003 [59]
Thyroid	NA	PC C13, RET/PTC1, PC E1A, COS-7	Transformed rat thyroid cell lines, Human thyroid DNA array	NA	Laukkanen 2010 [41]
Thyroid	Stromal SOD3 increased cancer cell growth	Mesenchymal stem/stromal cells isolated from human papillary thyroid cancer	qPCR expression of SOD3 measured in normal thyroid stromal cells and papillary thyroid cancer stromal cells	Increased SOD3 expression in cancer mesenchymal stem/stromal cells vs. normal MSCs	Parascandolo 2017 [42]
Ovarian	NA	SKOV-3, MDAH-2774	In vitro*—*DCA administration, shifting cell metabolism from anaerobic to aerobic	Increased apoptosis and increased EcSOD	Saed 2010 [60]
Ovarian	NA	SKOV-3 MDAH-2774	In vitro*—*DCI administration, inhibiting NADPH oxidase production of ROS	Increased apoptosis and decreased EcSOD	Jiang 2011 [61]
Renal	Increased apoptosis and proliferation with higher EcSOD expression	Human RCC samples	NA	NA	Soini 2006 [43]

## Data Availability

All data are contained within the manuscript.
